# Dynamic Imaging of CD8^+^ T Cells and Dendritic Cells during Infection with *Toxoplasma gondii*

**DOI:** 10.1371/journal.ppat.1000505

**Published:** 2009-07-03

**Authors:** Beena John, Tajie H. Harris, Elia D. Tait, Emma H. Wilson, Beth Gregg, Lai Guan Ng, Paulus Mrass, David S. Roos, Florence Dzierszinski, Wolfgang Weninger, Christopher A. Hunter

**Affiliations:** 1 Department of Pathobiology, School of Veterinary Medicine, University of Pennsylvania, Philadelphia, Pennsylvania, United States of America; 2 Division of Biomedical Sciences, University of California, Riverside, California, United States of America; 3 Department of Biology, University of Pennsylvania, Philadelphia, Pennsylvania, United States of America; 4 The Wistar Institute, Philadelphia, Pennsylvania, United States of America; 5 The Centenary Institute for Cancer Medicine and Cell Biology, Newtown, New South Wales, Australia; 6 Institute of Parasitology, McGill University, Montreal, Quebec, Canada; London School of Hygiene and Tropical Medicine, United Kingdom

## Abstract

To better understand the initiation of CD8^+^ T cell responses during infection, the primary response to the intracellular parasite *Toxoplasma gondii* was characterized using 2-photon microscopy combined with an experimental system that allowed visualization of dendritic cells (DCs) and parasite specific CD8^+^ T cells. Infection with *T. gondii* induced localization of both these populations to the sub-capsular/interfollicular region of the draining lymph node and DCs were required for the expansion of the T cells. Consistent with current models, in the presence of cognate antigen, the average velocity of CD8^+^ T cells decreased. Unexpectedly, infection also resulted in modulation of the behavior of non-parasite specific T cells. This TCR-independent process correlated with the re-modeling of the lymph node micro-architecture and changes in expression of CCL21 and CCL3. Infection also resulted in sustained interactions between the DCs and CD8^+^ T cells that were visualized only in the presence of cognate antigen and were limited to an early phase in the response. Infected DCs were rare within the lymph node during this time frame; however, DCs presenting the cognate antigen were detected. Together, these data provide novel insights into the earliest interaction between DCs and CD8^+^ T cells and suggest that cross presentation by bystander DCs rather than infected DCs is an important route of antigen presentation during toxoplasmosis.

## Author Summary

*Toxoplasma gondii* is a protozoan parasite that can infect a wide range of hosts, including humans. Infection with *T. gondii* is potentially life threatening in immuno-compromised individuals and it can be detrimental during pregnancy, often leading to abortion of the fetus. Dendritic cells are thought to play a vital role in the development of protective immunity to *Toxoplasma gondii* through their ability to produce immunological signals such as cytokines and also process and present parasite derived peptides to T cells. However, little is known about the actual interactions between these cell types in an intact organ, such as the lymph node, during infection. Using the technology of live imaging by 2-photon microscopy we have identified a very early window of time during infection when dendritic cells and T cells make sustained contacts with one another, which appears crucial for the generation of protective responses. We also show that substantial changes are induced in the lymph node micro-architecture as a result of infection, which in turn could have effects on immune responses to secondary pathogens. Understanding the interaction between these immune cells *in vivo* that leads to resistance to active infection would help in the design of better strategies to develop protective immune responses against this pathogen in immuno-compromised individuals.

## Introduction

*Toxoplasma gondii* is an intracellular protozoan parasite that induces a type 1 immune response characterized by the production of IFN-γ from CD4^+^ and CD8^+^ T cells [1],[2],[3]. The generation of this protective T cell response is dependent on the early synthesis of IL-12 by innate immune cells such as DCs, macrophages and neutrophils [4],[5]. Of these populations, DCs appear to have a central role in bridging innate and adaptive responses and mice depleted of DCs are more susceptible to *T. gondii* [6]. This phenotype has been linked to the reduced IL-12 production in these mice, although DCs also act as antigen presenting cells for T cell priming. Indeed infection with *T. gondii*, results in an increase in the total numbers of DCs, their activation status (increase in levels of MHC I, MHC II, CD80 and CD86) and changes in subset composition [7]. It is unclear from the earlier studies whether the depletion of DCs increases susceptibility to acute toxoplasmosis primarily through reduced IL-12 synthesis or by secondary effects on antigen presentation and T cell priming [6]. In addition, non-hematopoietic cells are also infected by *T. gondii* and can prime CD8^+^ T cells [8]. This observation has led to questions about the relative contribution of DCs and other antigen presenting cells in shaping the early T cell response during infection with *Toxoplasma*. While there is considerable evidence that CD8^+^ T cells and DCs are required for the control of *T. gondii* [3],[9], the actual interactions between these cells *in vivo* during toxoplasmosis have not been characterized.

Live imaging by 2-photon microscopy combined with the generation of transgenic mice expressing fluorescent tags specific for different immune cell populations, has enabled the visualization and tracking of these cells in real time within primary and secondary lymphoid organs [10]–[16]. The dynamics of T cell movement within the lymph nodes have been extensively characterized using 2 photon microscopy [17]–[22]. This has led to a model whereby naïve T cells survey lymph nodes, guided by fibroblastic reticular cell networks and localized expression of chemokines which promote the chances of interaction between a rare antigen specific T cell and an APC carrying its cognate antigen [10],[18]. However, many of the pioneering studies using intravital imaging, that have been used as a benchmark for understanding T cell behavior, have largely been based on non-infectious models [17], [19]–[24]. More recent studies have imaged the response of immune cells to pathogens [25]–[30] and some differences have emerged between the infectious and non-infectious systems. For example, when antigen pulsed DCs were used to prime adoptively transferred T cells, the T cells and DCs were largely limited to the T cell zones of the lymph node [11],[17],[20]. In contrast, challenge with vaccinia or vesicular stomatitis virus has shown the presence of viral antigens, dendritic cells and T cells within the sub-capsular and interfollicular regions of the lymph nodes [29],[30]. Thus there is value to being able to compare the mechanics of these events using reductionist approaches and models of infection.

One of the obstacles to understanding the early events during toxoplasmosis has been the inability to reliably identify the cells responding to *T. gondii* in tissues. The availability of transgenic parasites that express model antigens and the use of TCR transgenic adoptive transfer systems has now improved our ability to track the antigen specific CD8^+^ T cell response during various infections including those caused by *Toxoplasma*, *Listeria and Leishmania* [8], [31]–[34]. Recently the dynamics of activated OT1^GFP^ cells responding to *Toxoplasma* expressing ovalbumin, has been characterized in the brain by live imaging [35] but these approaches have not yet been applied to understand the early events of T cell priming during this infection [32].

As a part of studies to better understand how T cell mediated immunity to *T. gondii* is initiated, transgenic *Toxoplasma* parasites and fluorescent immune cells were combined to enable live imaging by 2-photon microscopy of parasite specific CD8^+^ T cells and their interactions with DCs. These studies show that early during infection there was significant recruitment of dendritic cells and T cells to the sub-capsular region of the lymph node and that DCs were required for the expansion of T cells. In the presence of cognate antigen there was a significant reduction in the CD8^+^ T cell velocity, however infection by itself also had a non-specific effect on T cell movement. These latter events correlated with alterations in lymph node architecture and reduced expression of CCL21, a chemokine that provides motogenic signals that underlie T cell movement in lymphoid tissues. Infection also led to a significant increase in CD8^+^ T cell-DC contacts in the presence of cognate antigen; however, sustained interactions were limited to a very early time point during infection, which correlated with the presentation of antigen by DCs. While infected DCs were rare at these early time points, DCs that were capable of presenting the cognate antigen were detected. Together, these studies reveal several novel aspects of CD8^+^ T cell and dendritic cell behavior and suggest that cross-presentation has a role in the development of protective T cell responses during toxoplasmosis.

## Results

### Kinetics of OT1 CD8^+^ T cell response to *Toxoplasma* infection

**Figure 1:**
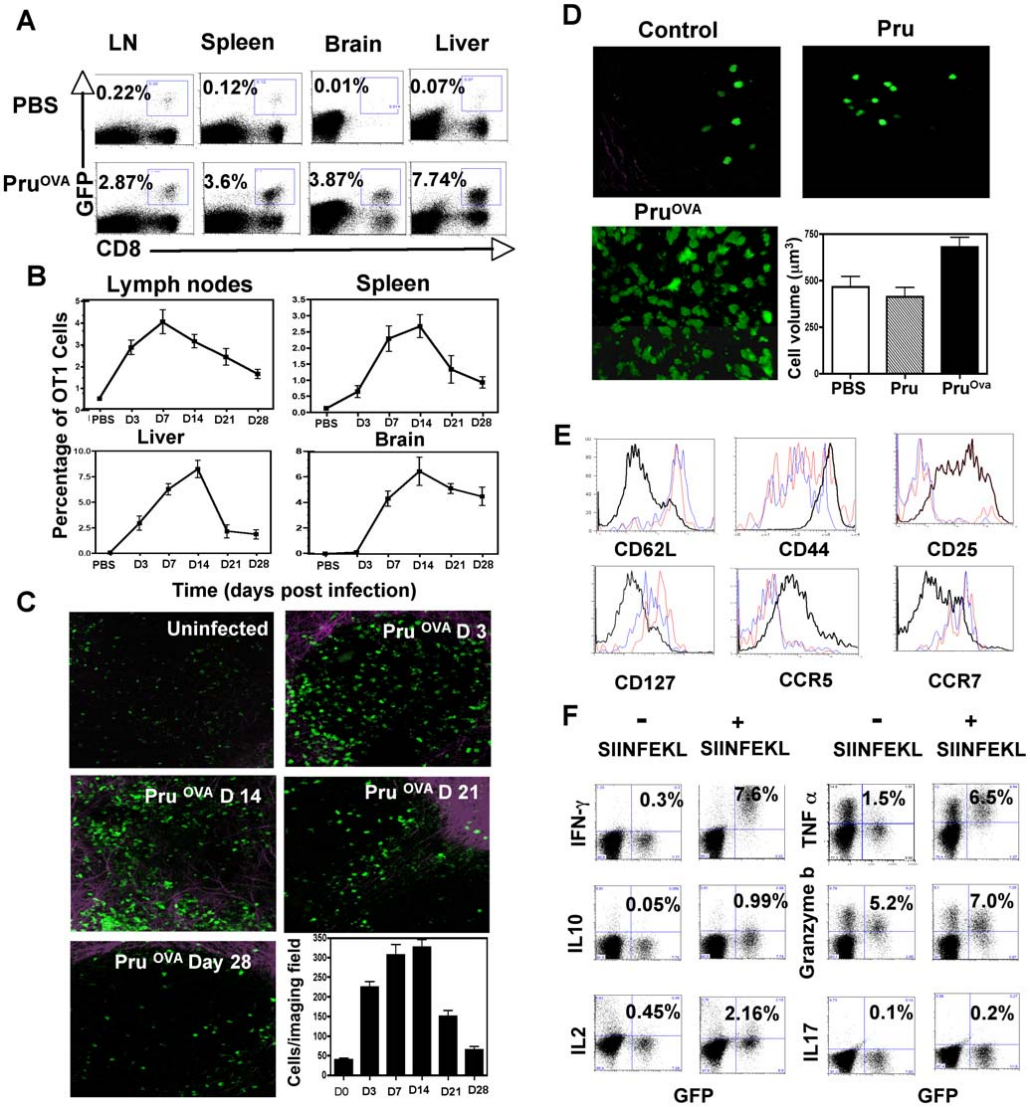
OT1^GFP^ T cell response to *Toxoplasma* infection. A) The frequency of transferred OT1^GFP^ cells in the spleen, mesenteric lymph nodes, brain and liver of uninfected and Pru^OVA^ infected mice (Day 7 post infection). B) The average frequency of OT1 cells (percentage of live cells) at different time points post infection in the lymph nodes, spleen, liver and brain of mice infected with Pru^OVA^ parasites (n = 4 per time point). C) Maximum focus images from 30 µm stacks of mesenteric lymph nodes of Pru^OVA^ infected mice at various time points post infection (resolution 0.98 µm/pixel). OT1^GFP^ cells are in seen in green and the second harmonic signals generated by the lymph node capsule are seen in purple. D) Representative snapshots of OT1^GFP^ cells imaged in the mesenteric lymph nodes of uninfected mice (control) and mice infected with Pru or Pru^OVA^ parasites (day 3-post infection). The average volume of the cells that were imaged in the three different groups of mice is also shown. E) The expression of various surface receptors CD62L, CD44, CD25, CCR7, CCR5 on the OT^GFP^ cells (GFP^+^ CD8^+^) from uninfected mice (red) and mice infected with Pru (blue) or Pru^OVA^ (black) at day 7 post infection. F) Intra-cellular cytokine profile (IFNγ, IL-2, IL-10, TNFα, Granzyme-B, IL-17) of gated CD8^+^ T cells from the lymph nodes of Pru^OVA^ infected mice (day7 post infection) that were either unstimulated or stimulated with SIINFEKL peptide for 5 hours *ex vivo* in the presence of Brefeldin A.

In order to visualize the CD8^+^ T cell response during *Toxoplasma* infection, a model was used in which naive T cells from OT1^GFP^ mice [36] were transferred into recipients, which were then infected with a genetically modified strain of *T.gondii* expressing ovalbumin (Pru^OVA^). As a first step in these studies, we quantified the OT1^GFP^ response during the course of infection by flow cytometry. In uninfected mice, the transferred OT1^GFP^ cells were present in all tissues except the brain (Figure 1A). As expected, infection with Pru^OVA^ resulted in an expansion of these cells over the first 14 days in all compartments (Figure 1B). The response was followed over a period of 28 days and the OT1^GFP^ cells showed a gradual contraction in the spleen, mesenteric lymph nodes and liver following the initial expansion. Our studies did not reveal any difference between the mesenteric, mediastinal and parathymic lymph nodes either in numbers of OT1 T cells recovered or their activation phenotypes, consistent with recent reports which indicate that all of these lymph nodes can drain the peritoneal cavity [37]. The only site that displayed a differential kinetics was the brain, where there was a delay in the appearance of the OT1^GFP^ cells. The response was maintained in the brain over the time frame analyzed consistent with parasite persistence at this site (data not shown). The numbers of cells that were detected at various time points by 2-photon microscopy within the mesenteric lymph node was consistent with the kinetics established by flow cytometry (Figure 1C).

As an integral part of these studies, it was important to determine whether infection on its own affected the phenotype and behavior of the transferred cells. Therefore, mice adoptively transferred with OT1^GFP^ cells were infected with the parental strain of *Toxoplasma* (Pru) or with the ovalbumin expressing strain (Pru^OVA^) and the phenotype of the OT1^GFP^ cells was compared. The OT1^GFP^ cells imaged by 2-photon microscopy within the lymph nodes of uninfected or Pru infected mice showed a naive phenotype based on their smaller size and total numbers (Figure 1D). The mice challenged with Pru^OVA^ however showed clonal expansion of the transferred OT1^GFP^ cells and these cells showed a 30–40% increase in cellular volume over OT1^GFP^ cells in the uninfected or Pru infected mice (Figure 1D). Analysis of activation markers revealed that the OT1^GFP^ cells in Pru infected mice retained their naive phenotype (CD62L^hi^ CD127^hi^ CD44^lo^ CD25^lo^) comparable to the OT1^GFP^ cells in uninfected mice (Figure 1E). In contrast, OT1^GFP^ cells from the Pru^OVA^ infected mice (day 7 post infection) displayed an activated phenotype (CD62L^lo^ CD127^lo^ CD44^hi^ CD25^hi^). The chemokine receptor expression on the surface of these cells also changed upon activation. The OT1^GFP^ cells in the PBS and Pru infected mice were CCR7^hi^ CCR5^lo^ in contrast to OT1^GFP^ cells in Pru^OVA^ infected mice, which were CCR7^lo^ CCR5^hi^.

To determine whether these transferred cells acquire effector functions typical of the CD8^+^ T cell response to *T.gondii*, a 5-hour *ex-vivo* restimulation assay was performed. In the infected mice (day 7 post infection), upon restimulation with SIINFEKL peptide, the OT1^GFP^ cells synthesize effector cytokines such as IFN-γ, TNF-α and expressed increased levels of granzyme-B indicating that they are poised for cytolysis (Figure 1F). The OT1 cells in the Pru infected mice did not synthesize any of these effector cytokines during ex-vivo restimulation with SIINFEKL (Figure S1). These data show that the OT1^GFP^ cells that expanded in the lymph nodes in response to infection with Pru^OVA^ were antigen specific and fully functional effectors.

### Visualization and analysis of the CD8^+^ T cell behavior in response to *T. gondii* infection

**Figure 2:**
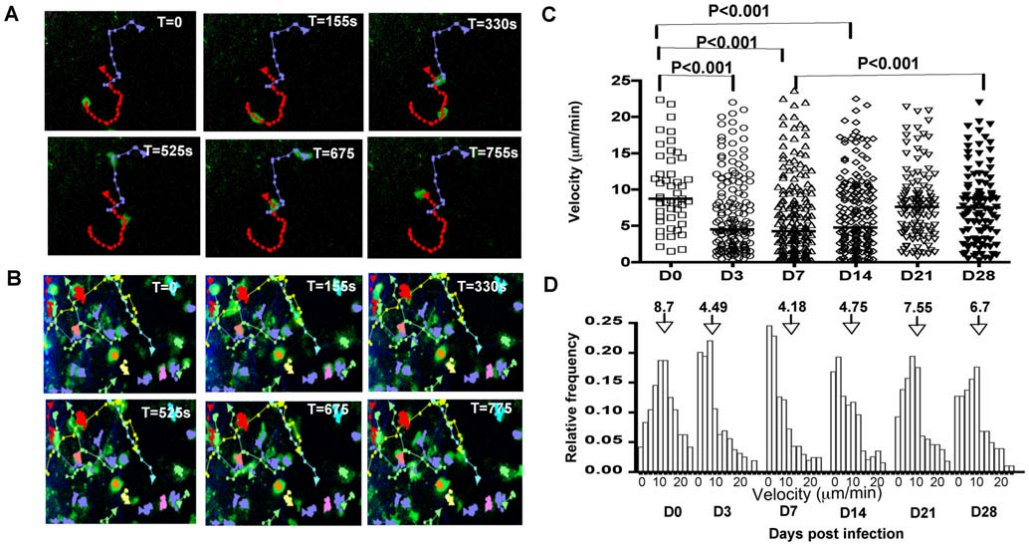
Antigen dependent reduction in OT^GFP^ cell velocities during *Toxoplasma* infection. A) Tracks followed by OT1^GFP^ cells during a given imaging session (total time = 755 s) in the mesenteric lymph node of an uninfected mouse. The OT1^GFP^ cells are shown in green and the tracks generated by these cells are shown in red and purple. B) T cell tracks from mice infected with Pru^OVA^ (D3 post infection) during an imaging session lasting 755 s. The OT1^GFP^ cells are shown in green and the cell tracks are represented in multiple colors C) The mean migratory velocities of the imaged cells at the different time points post infection. Each symbol corresponds to a tracked T cell and bars indicate median velocity of the populations. P values were calculated using Kruskal-Wallis test. D) The frequency of the OT1^GFP^ cells moving at various velocities as a function of the total imaged cells, at the different time points post infection. Arrows point to the median velocity of each population.

Having established that ova-expressing parasites induce a relevant response in OT1^GFP^ cells, live imaging by 2-photon microscopy was used to visualize the behavior of these CD8^+^ T cells. Following T cell transfer, mice were either infected with Pru^OVA^ or left uninfected. Mesenteric lymph nodes were isolated from the mice at different times post-infection and explanted lymph nodes were imaged using a temperature controlled, perfused imaging chamber. Tracking of individual T cells was performed to determine how infection-induced activation affects cell velocity, displacement and meandering index. The tracks of naive and activated OT1^GFP^ cells at various time points (0 s, 155 s, 330 s, 525 s, 675 s and 755 s) during an imaging session (day 3 post infection) is shown in Figure 2 (A&B). Analysis of the mean migratory velocities of the T cells during the entire imaging period revealed differences between the naive and activated OT1^GFP^ cells (Video S1). Naive T cells moved with an average velocity of 8.7 µm/min and as early as 3 days post-infection with Pru^OVA^, there was a reduction in the average velocity of the population to 4.49 µm/min (Figure 2C). A similar reduction was also seen at days 7 and 14 post infection (4.18 µm/min and 4.75 µm/min respectively), however at the later time points (day 21 and day 28) the average velocities increased to 7.5 and 6.7 µm/min respectively.

While infection resulted in a statistically significant reduction in the average velocity of the transferred T cells, it was apparent that at all time points examined, there was a large range in motility: fast (15–20 µm/min) and slower moving cells (5–10 µm/min) and a population with a highly constrained phenotype (0–2 µm/min). To provide a more complete analysis of the changes in T cell behavior, the frequencies of cells that move at different speeds within the given imaging session are shown in Figure 2D. Presenting the data in this fashion revealed that by day 3 following challenge with Pru^OVA^, the reduction in the average T cell velocity was largely a function of the increased proportion of cells that were moving at less than 2 µm/min. At day 7 and 14, this population remains the largest fraction, but by day 21 the distribution of T cell velocities starts to revert back to the normal distribution seen in uninfected mice. Thus, during the early stages of infection within the lymph node, there is an increase in the proportion of stationary cells, which is reversed at later time points.

### Antigen dependent and antigen independent effects of infection on T cell movement

**Figure 3:**
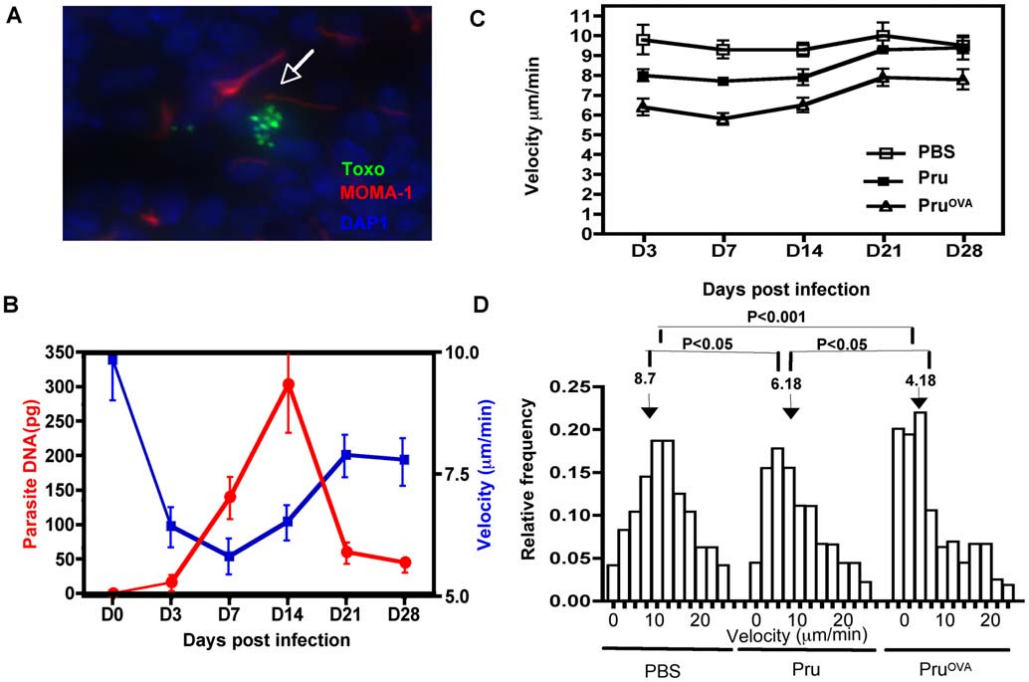
Antigen specific and antigen non-specific effects of infection on T cell motility. A) Immuno-histochemical staining for *T.gondii* (green) DAPI (blue) and MOMA-1 (red) in an infected lymph node. B) The correlation between parasite DNA levels (red) measured by RT-PCR from the mesenteric lymph nodes of Pru^OVA^ infected mice and average OT1^GFP^ T cell velocity (blue), at various time points post Pru^OVA^ infection. C) The average velocity of OT1^GFP^ cells calculated from tracks generated from time-lapse imaging of lymph nodes from uninfected, Pru infected and Pru^OVA^ infected mice at various time points post infection. D) The frequency of the OT1^GFP^ cells moving at various velocities as a function of the total imaged cells, in the different immune groups on day 7 post infection. P values were calculated using Kruskal-Wallis test.

In other models, pausing and stalling of T cells is associated with recognition of cognate antigen and activation. Since *T.gondii* infects DCs and macrophages and disseminates widely throughout the host, it seemed likely that antigen availability contributed to the changes observed in T cell behavior. Parasites can be visualized, albeit at low numbers, by immuno-histochemical staining within the lymph node (Figure 3A) and measurement of parasite DNA by real time PCR revealed an initial increase followed by a decline in the parasite DNA levels (Figure 3B). Interestingly, while the decrease in T cell velocities was inversely correlated with parasite burden, the maximal reduction in the T cell velocities preceded the peak of parasite burden in these tissues (Figure 3B).

In order to determine whether infection on its own affects T cell movement in the absence of antigen, OT1^GFP^ cells from uninfected, Pru and Pru^OVA^ infected mice were compared. The virulence of the two different strains were comparable as noted in previous studies [38] and as shown by the parasite DNA levels measured in the lymph nodes of mice infected with either Pru or Pru^OVA^ (Figure S1B). Analysis of OT1^GFP^ cells in Pru infected mice (no cognate antigen) revealed a modest and transient reduction in the average velocity in comparison to OT1^GFP^ cells in uninfected mice (Figure 3C). However, unlike the OT1^GFP^ cells in the Pru^OVA^ infected mice, the reduction in the average velocity of the population was not due to a preponderance of cells that had stopped, but due to an increase in the proportion of slower moving cells (Figure 3D). These latter data indicate that while antigen availability plays a vital role in CD8^+^ T cell movement within the lymph node, there are antigen independent effects on T cell motility during this infection.

### Infection induced remodeling of lymph node architecture and changes in the chemokine expression

**Figure 4:**
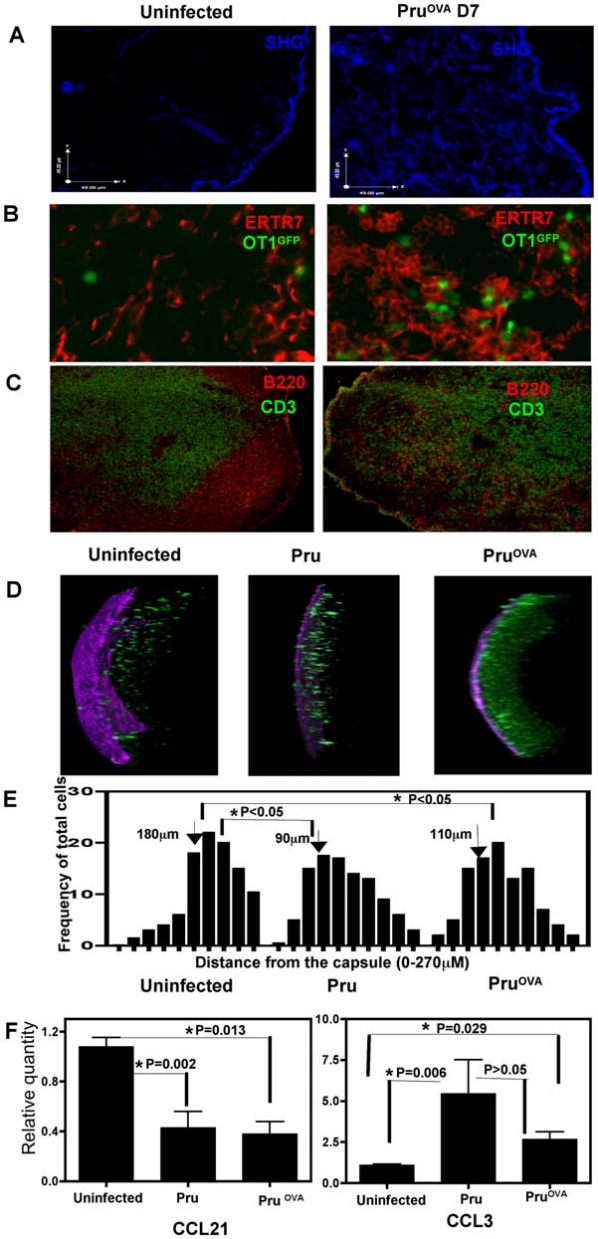
Changes in lymph node architecture during infection. A) The second harmonic signals in lymph node sections (6 µm) from uninfected and infected mice (Day 7 post infection). B) Immuno-staining for ERTR7 in lymph nodes sections from uninfected or Pru^OVA^ infected mice. OT1^GFP^ cells can be visualized in green C) Staining for CD3 (green) and B220 (red) from 6 µm sections of lymph nodes from uninfected or infected mice. D) 3D projections of 300 µm z stacks obtained from lymph nodes of uninfected, Pru and Pru ^OVA^ infected mice (day 5 post infection). E) The average frequency of cells at different distances (0–270 µm) from the capsule (location of the capsule = 0 µm). F) Levels of CCL21 and CCL3 measured by RT-PCR, normalized to control HPRT levels and expressed relative to the naive uninfected controls. The test samples were from mesenteric lymph nodes of mice infected with Pru or Pru^OVA^ 7 days prior. Bars indicate SEM (n = 4 per group).

In order to understand the antigen independent modulation of T cell movement during infection, studies were performed to determine if there were any changes in the microenvironment of the infected lymph node and the localization of OT1^GFP^ cells. Collagen fibrils have been shown previously to underlay the conduit system formed by the fibroblastic reticular cell networks within the lymph node [17] and collagen is known to generate second harmonic signals during imaging by multiphoton microscopy. To visualize these structures, lymph node sections were exposed to polarized laser light (930 nm) and the second harmonic structures were detected using non-descanned detectors with barrier filters in the 457–487 nm range. In response to infection, there was a marked increase in the second harmonic structures within the lymph node (Figure 4A). Quantification of the volume of fibers visualized at day 7 reveals an almost 2 fold increase in these networks (data not shown). Immuno-histochemical staining of lymph node sections for ERTR7, a marker for fibroblastic reticular cells (FRC). revealed that infection resulted in an increase in their density (Figure 4B). (The second harmonic signals generated in the lymph node show co-localization with the ERTR7 staining (Figure S2A)). In a naïve lymph node, the ERTR7 networks are confined to the T cell zones, but this well-defined organization is absent in the lymph nodes of the infected mice. This is in agreement with the loss of distinct T cell areas and B cell follicles upon infection (Figure 4C). Differences were also seen in the localization of the OT1^GFP^ cells relative to the capsule in the infected and uninfected mice. This is depicted in the representative z-stacks (20×) of the mesenteric lymph nodes from the three different experimental groups (Figure 4D). In uninfected mice, the majority of OT1^GFP^ cells are located away from the capsule, whereas infection leads to the presence of OT1^GFP^ cells in the sub-capsular region. This is reflected in the average frequency of the cells imaged at different distances from the capsule (seen in purple) from 300 µm z stacks of lymph nodes from the various treatment groups (Figure 4E). This change in the localization of the OT1^GFP^ cells was independent of the presence of cognate antigen as it was also observed in Pru infected mice.

The chemokine environment within the lymph node, specifically the balance between CCL21/CCL19 and pro-inflammatory chemokines such as CCL3, has been linked to T cell motility and their directionality [10],[21]. Therefore, mRNA levels for CCL21 and CCL3 within the lymph node, during infection were measured by RT-PCR. Compared to uninfected mice, CCL21 expression (normalized against control HPRT expression) showed a down-regulation in the infected lymph nodes, while CCL3 expression showed a modest increase (Figure 4F). Preliminary studies did not reveal significant changes in the levels of CCL5 in the lymph node during the time points when changes in CCL3 and CCL21 were observed (data not shown). As noted earlier, during infection with Pru, the naive OT1^GFP^ cells retain high levels of CCR7 and do not up regulate CCR5 (Figure 2B). Thus, while the balance between the chemokines CCL21 and CCL3 changes during infection, the receptors for these chemokines, CCR7 and CCR5 respectively, are not altered on the naive T cells in the absence of cognate antigen. Together, these data show that infection induces distinct changes in the location of the T cells and micro architecture of the lymph node, all of which may contribute to the antigen independent regulation of T cell movement.

### Role of DCs during early infection

**Figure 5:**
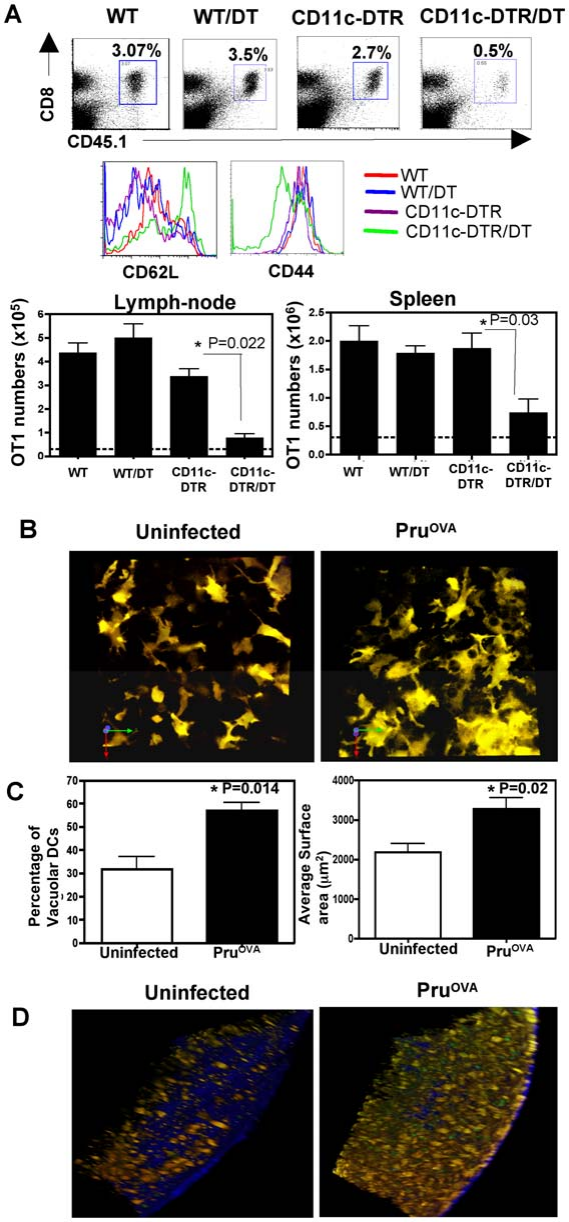
Role of DCs during infection with *Toxoplasma*. A) The percentages of OT1^GFP^ cells (CD45.1^+^CD8^+^) in WT or CDllc-DTR transgenic mice that were infected (day 5-post infection) with Pru^OVA^ with or without treatment with DT (100 ng/mouse ip). The CD62L and CD44 levels on the OT1^GFP^ cells (CD45.1^+^CD8^+^T cells) present in the lymph nodes of mice from the various treatment groups. The average OT1 numbers from spleen and pooled lymph nodes of 3 mice per group is shown in the lower panels. The line represents the average OT1 numbers in uninfected WT mice. Similar results were obtained in a separate experiment. B) Maximum focus images of z stacks (21 µm) from draining lymph nodes of CD11c^YFP^ mice that were either uninfected or infected (Pru^OVA^) 3 days prior. C) The average number of vacuolar DCs and the average surface area of the DCs in infected and uninfected groups. D) 3D projections of 150 µm z stacks from lymph nodes of CD11c^YFP^ transgenic mice that were adoptively transferred with OT1^GFP^ T cells and were either uninfected or infected with Pru^OVA^ (day 3-post infection).

Since infection with Pru^OVA^ induced a significant proportion of the OT1^GFP^ cells to arrest and round up very early during infection, it seemed likely that this was a reflection of cross talk with professional APCs such as DCs. Previous studies have indicated that in the absence of DCs mice are susceptible to infection with *T.gondii* and this was linked to reduced IL-12 production in these mice [6]. In order to test whether transient depletion of DCs had any effect on T cell priming we used the CD11c-DTR transgenic model [39]. The CD11c-DTR transgenic mice express the diphtheria toxin (DT) receptor under the control of CD11c promoter and hence transient depletion of CD11c^+^ cells (2 days) is achieved by injecting DT into these mice. CD11c-DTR^GFP^ transgenic or WT mice received OT1^GFP^ cells, followed by injection of DT (100 ng/mouse). The mice were then infected with Pru^OVA^ parasites 24 hours later. Transient depletion of DCs was verified in the CD11c-DTR^GFP^ transgenic mice by flow-cytometric analysis of peripheral blood samples 24 hours after treatment with DT (data not shown). The frequency and numbers of OT1^GFP^ cells that were detected in the spleen and lymph nodes were significantly reduced in the CD11c-DTR^GFP^ transgenic mice treated with DT compared to the CD11c-DTR^GFP^ mice that were untreated or WT mice that were treated with DT (Figure 5A). A large fraction of the OT1^GFP^ cells recovered from the CD11c-DTR^GFP^ transgenic mice treated with DT retained a naïve phenotype (CD44^lo^CD62L^hi^). There was however a small proportion of OT1^GFP^ cells that showed an activated phenotype CD44^hi^CD62L^lo^. These studies indicate that DCs are the major APCs for priming a CD8^+^ T cell response and their presence is crucial early during infection to generate an efficient CD8^+^ T cell response.

In order to visualize if infection induced changes in the DC population, transgenic mice in which the CD11c promoter drives expression of YFP were used [40]. In uninfected mice, the CD11c^YFP^ cells present in the lymph node displayed a morphology typical of DCs described previously [40] (Figure 5B, Videos S2 and S4). Upon infection, these cells developed a highly vacuolated appearance and showed an increase in their surface area (day 3 post infection, Figure 5C). It should be noted that while a large proportion of DCs displayed this vacuolar phenotype, the number of infected cells present in these tissues was low. Further, in the rare instance when an infected and an uninfected DC could be visualized within the same imaging field in the lymph node, both the DCs were vacuolated (Figure S3). This phenotype could be detected as early as 24 hours post infection, and persisted at days 7 and 14. Our studies did not reveal any significant differences in the movement of the DCs (Video S2) between infected and uninfected mice.

To assess whether the DCs and CD8^+^ T cells co-localize and interact, 2×10^6^ OT1^GFP^ cells were transferred into CD11c^YFP^ transgenic mice, which were then either infected with 10^4^ Pru^OVA^ parasites or left unchallenged. Distinct differences were noticed in the localization of the DCs (similar to the localization of OT1^GFP^ cells noted previously) during infection. In uninfected mice, the DCs were distributed largely in the T cell areas, B cell follicles and a small proportion can be found in the sub-capsular/interfollicular regions of the lymph nodes (data not shown). In mice infected with Pru^OVA^ there was a significant increase in the numbers of CD11c^YFP^ cells and OT1^GFP^ cells in the sub capsular/interfollicular region of the lymph node, as seen in the 150 µm z stacks (20×) at day 3 post infection (Figure 5D and Videos S3 and S4). This increased localization of DCs and CD8^+^ T cells to this region was also seen at days 5 and 7 post infection, but by day 28, the lymph node architecture was more similar to that observed in the uninfected state. Together these data reveal that infection with *Toxoplasma* leads to changes in DC morphology and localization within the lymph nodes draining the site of infection.

### DC-T cell interactions

**Figure 6:**
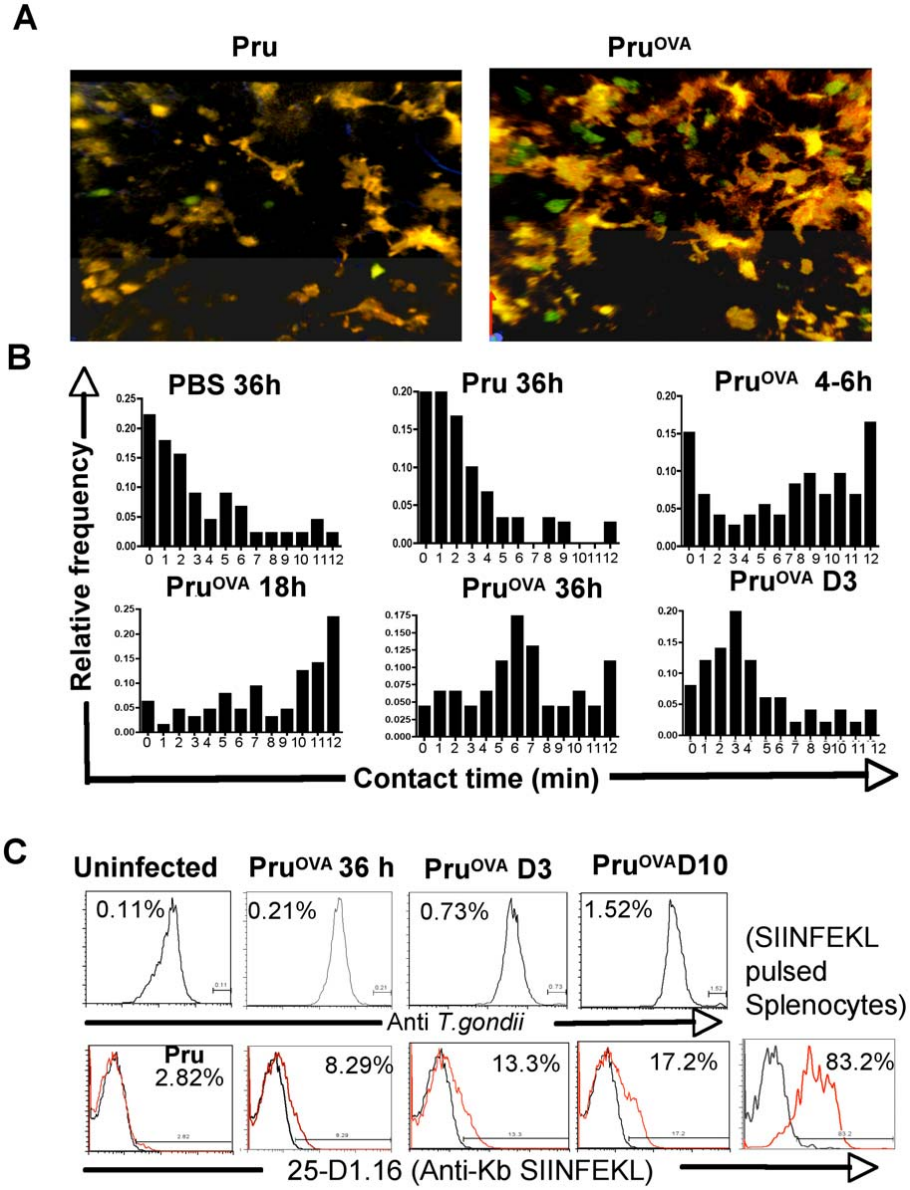
DC-T cell interactions. A) Maximum focus images of 30 µm stacks showing CD11c^YFP^ cells and OT1^GFP^ cells in Pru or Pru^OVA^ infected mice shown as a representative for the data used to quantitate the interactions between T cells and DCs. B) The relative frequency of OT1^GFP^ T cells interacting with CD11c^YFP^ cells and the duration of the contacts in uninfected, Pru infected and Pru ^OVA^ infected mice at 6, 18, 36 and 72 hours post infection is shown. The x-axis shows the T cell-DC contact time within the given imaging session and the y-axis shows the frequency of total OT1^GFP^ T cells visualized. Data obtained from an average of 6 imaging regions from 3 individual mice per group were used to obtain the frequency of T cells with different durations of contacts with the DCs. C) Intracellular staining for *T. gondii* (polyclonal rabbit anti *T.gondii* antibody) on CD11c+ cells (top panel). Staining with 25-D1.16 (bottom panel) on CD11c+ cells from Pru or Pru^OVA^ infected mice (red) at various time points post infection compared to the uninfected mice (black). SIINFEKL peptide (30 µM) pulsed splenocytes were used as a positive control for staining with 25-D1.16.

Since DCs and T cells localize to the same regions during infection, we wanted to determine if there were any antigen dependent crosstalk between these two populations. T cell-DC interactions were analyzed at different times post infection in the CD11c^YFP^ transgenic mice that were adoptively transferred with OT1^GFP^ cells. A representative image of DCs and OT1^GFP^ cells imaged during Pru and Pru^OVA^ infection is shown in Figure 6A. The frequency of cells that made contacts of different durations with DCs within the imaging sessions (typically 12–15 minutes) is shown in Figure 6B (Videos S5 and S6). In uninfected mice, OT1^GFP^ and CD11c^YFP^ cells had very brief encounters and infection with Pru did not lead to significant changes in these interaction times. In the presence of cognate antigen (Pru^OVA^) long-lived interactions could be observed as early as 6 hours post infection. However, at these early times, there was a large variation in DC-T cell interaction times as seen in Figure 6B. The frequency of T cells making prolonged contacts with DCs increases between 18–24 hours and most of the contacts could be visualized for the entire imaging period. Consequently, the upper limit of interaction times shown in Figure 6B is an underestimate of how long these two populations remain in contact. By 36 hours post infection with Pru^OVA^, in addition to the T cells that made long lasting interactions, there was a proportion of T cells that were engaged with DCs for comparatively shorter time periods. By day 3, however the majority of the interactions visualized were of substantially shorter durations (Videos S5 and S6). There were no significant changes in DC-T cell interaction times in the absence of cognate antigen (during Pru infection) during any of these time points. These data indicate that sustained interactions between CD8^+^ T cells and DCs are visualized only in the presence of cognate antigen and are most frequent very early during infection (within the first 36–48 hours). These sustained early interactions are antigen dependent, and are suggestive of antigen presentation by the dendritic cells to the OT1^GFP^ cells.

In order to ascertain whether the DCs that were interacting with the OT1^GFP^ T cells were infected, CD11c^YFP^ transgenic mice were infected with a Pru^OVA^ strain that was engineered to express cytoplasmic dTomato. This enabled the visualization of the parasites during live imaging. In the first 36 hours infected DCs were rarely detected in the mesenteric and mediastinal lymph nodes by 2-photon microscopy (data not shown). Intracellular staining with a polyclonal anti-*Toxoplasma* antibody similarly showed very few infected DCs (Figure 6C). However, staining with 25-D1.16 antibody, which recognizes MHC class I (K^b^)-SIINFEKL complexes [41], reveals that many of these cells were presenting the immunodominant peptide of ovalbumin. Immuno-staining for ovalbumin protein similarly showed DCs carrying ovalbumin early during infection (Figure S2B). These data suggest that the prolonged interactions that were visualized early during infection most probably involved DCs cross-presenting *Toxoplasma* derived antigens, rather than infected DCs.

**Figure 7:**
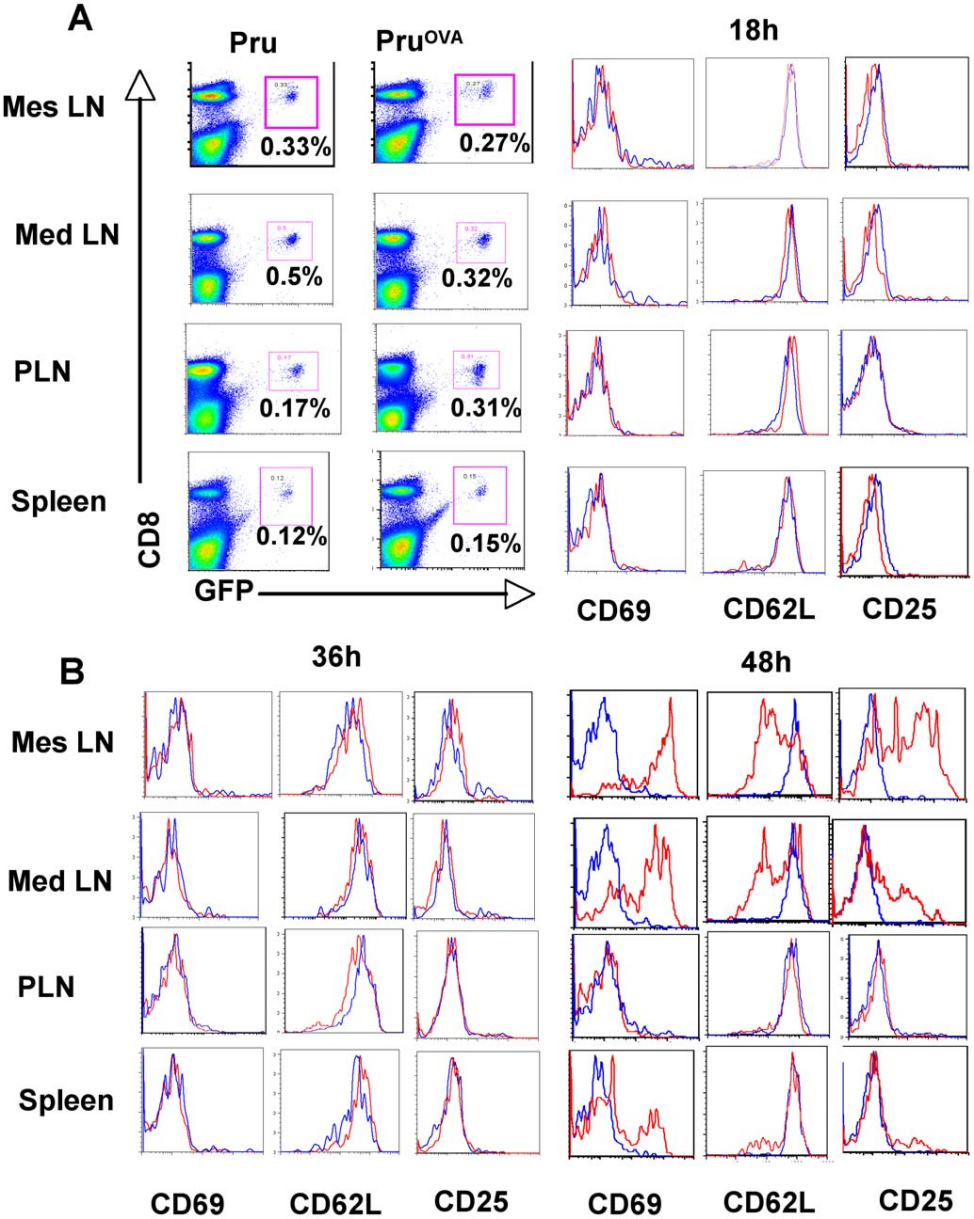
Early T cell activation kinetics. A) The percentage of OT1^GFP^ cells in the mesenteric, mediastinal, peripheral lymph nodes and spleen of mice infected with Pru or Pru ^OVA^ (right). The expression of activation makers (CD62L, CD69, CD25) on the OT1^GFP^ cells from the respective organs of Pru (blue) and Pru^OVA^ infected mice (red) at 18 hours post infection (left). B) Expression of the activation markers on the OT1^GFP^ cells from Pru (blue) or Pru^OVA^ infected (red) mice at 36 (left) and 48 hours (right) post infection.

The kinetics of activation of OT1^GFP^ cells early during infection was monitored in the mesenteric, mediastinal and peripheral lymph nodes as well as the spleen at various time points post-infection to see if the acquisition of activation markers mirrored the time frame seen with the DC-T cell interaction studies (Figure 7A). At 18 and 36 hours post infection the OT1 cells in all compartments retained their naïve phenotype (CD62L^hi^CD69^lo^CD25^lo^). By 48 hours, the OT1 cells in the mediastinal and mesenteric lymph nodes and a smaller proportion in the spleen showed signs of activation (CD62L^lo^CD69^hi^CD25^hi^) (Figure 7B). These studies indicate that the T cells start to express activation markers during the 36–48 hr time window subsequent to the initial prolonged interactions.

Together, these data show that the early-sustained interactions observed between DCs and T cells in the presence of cognate antigen are indicative of T cell priming.

## Discussion

With the advent of live *in vivo* imaging, significant advances have been made in our understanding of T cell behavior within secondary lymphoid tissues [12],[13]. While many of the early reports on this topic were based on non-infectious models, more recent studies have focused on the effects of inflammation and infection on immune cells in lymphoid and non-lymphoid compartments [11], [12], [20], [22], [25]–[30]. However, the dynamics of T-DC interactions during infection with a live replicating parasite have not been characterized previously and little is known about this process. The studies presented here describe the use of genetically modified parasites combined with TCR transgenic T cells and various reporters to allow visualization of *T. gondii* induced CD8^+^ T cell priming by DCs and the changes induced in these populations during infection.

Consistent with current models, the presence of cognate antigen was the major factor that influenced CD8^+^ T cell motility during toxoplasmosis. The stalling of the OT1^GFP^ cells observed in the presence of OVA expressing parasites could be due to a variety of reasons but the higher frequency of non-motile T cells very early during infection (36 hours) correlated with sustained contacts with DCs. In other reports, long lived interactions between DCs loaded with the antigenic peptide and CD4^+^ T or CD8^+^ T cells were imaged in the first 18–36 hrs after transfer of T cells [19],[22]. Moreover, in other infectious disease systems in which anti-bacterial drugs were used to limit bacterial replication, the first 24–48 hrs after challenge was crucial for T cell priming [42],[43]. Thus, placed in this context, our findings indicate that the CD8^+^ T cell priming events during *Toxoplasma* infection happen much earlier than previously anticipated.

Recent studies have dissected the priming response of CD8^+^ T cells into three stages: an initial phase dominated by transient short term interactions, followed by prolonged interactions and a third phase of short lived interactions between T cells and DCs [44]. While it is difficult to compare the results observed using DCs loaded with defined amount of peptide to an infection where antigen availability increases over time and the pathogen is modulating the host cell response, our studies show multiple stages in the T-DC crosstalk, with a transition from sustained interactions at the early time points to short lived interactions at the later time points. However, the duration of some of these T-DC contacts was difficult to estimate, since many of these lasted through out the imaging sessions. As infection progressed and T cells became activated, the interactions with DCs were of much shorter duration consistent with other reports [44],[45]. This change in behavior reflects the evolution of the T cell response from a naive resting population to one composed predominantly of effector cells which have an intrinsically lower threshold for activation [46] and perhaps short term interactions with DC are sufficient to sustain the expansion of the CD8^+^ T cell response.

One of the most notable changes following infection was in the organization of the lymph node associated with the presence of T cells and dendritic cells in the sub-capsular/interfollicular region. Similar reorganization was noted using vaccinia or vesicular stomatitis virus infections, where T cells and antigen were found largely in the sub-capsular region [29],[30]. The redistribution of T cells and DCs during challenge with *T. gondii* could be due to the entry of these cells from the afferent lymphatics and accumulation at this location. The other possibility is that with the first round of parasite cytolysis of infected cells, there would be the release of parasite-derived secreted antigens from the parasitophorous vacuole that would drain into the lymph node through the afferent lymphatics. The ability of parasite-derived material to mobilize DCs has been reported previously [47] and this might mediate active relocalization of DCs to this site. A related possibility is that parasites disseminate rapidly from the site of infection and studies by Robey and colleagues using a virulent strain of *T. gondii* that imaged the neutrophil response to *T. gondii* noted the presence of parasites and inflammatory cells within the sub-capsular region [25]. This suggests that this location is a site of active inflammation and/or parasite replication.

An unexpected finding from these studies was the antigen-independent modulation of CD8^+^ T cell motility, which correlated with the extensive remodeling of the lymph node during infection. In uninflamed lymph nodes, the FRC networks and the chemokines CCL19 and CCL21 that decorate these networks provide the major chemo-kinetic stimulus for the movement of naive T cells [18]. Changes in these structures have been reported in some infections, such as challenge with LCMV, where there is destruction of the FRC network [48] and down-modulation of CCL21 [49]. Similarly, challenge with *Toxoplasma* results in a disorganization of the FRC network, possibly due to the loss of well-defined T cell areas and B cell follicles, and a reduction in the levels of CCL21. Since, in the absence of cognate antigen, the OT1^GFP^ cells still maintain high levels of CCR7, it is possible that the reduced motogenic signals associated with loss of CCL21 combined with changes in the architecture of the lymph node account for the reduction in T cell motility. Whether this change in behavior would reduce the likelihood of a naïve T cell contacting an appropriate DC or if it represents a mechanism to promote the development of more sustained interactions and so promote the priming of naive T cells in an inflamed environment remains to be tested. However, there are multiple reports suggesting that it is difficult to prime new T cell responses during acute infection/inflammation [49],[50]. The phenomenon of antigen-independent slowing of naïve T cells in an inflamed environment observed in this study, may help to explain the basis for these previous observations.

In the current studies, significant changes were also observed in the morphology of the DCs in infected mice. The appearance of multiple vacuolar structures was the most characteristic change and the initial expectation was that these might contain parasites, similar to what has been reported for *Leishmania* parasites [26]. However, the number of vacuolated DCs that contained parasites was rare and the majority of vacuolated DCs were uninfected. However, vacuolation of the DCs was observed only during infection with live parasites and not by injection of soluble tachyzoite antigen (unpublished observations), suggesting that it is a consequence of the inflammatory events associated with infection. A similar morphological change has also been observed for astrocytes during toxoplasmic encephalitis (unpublished observations).

In current paradigms dendritic cells play a central role in the development of resistance to *T. gondii* likely through the production of IL-12 as well as antigen presentation. Moreover, recent studies from this laboratory and others, using a type I virulent strain of *T. gondii*, concluded that only infected DCs are capable of presenting antigens for the generation of CD8^+^ T cell responses [8],[51]. However, the studies presented here, using an avirulent type II strain of *T. gondii*, revealed early sustained interactions between OT1^GFP^ cells and DCs at a time point when the number of infected cells in the lymph node was minimal suggesting that uninfected DCs are capable of priming the CD8^+^ T cell response. Taken together, these results suggest that, depending on the parasite strain, there may be fundamentally different antigen sampling and/or processing pathways that dictate how the CD8^+^ T cell response to *T. gondii* is generated. Using available reporters and KO mice it should now be possible to distinguish how strains of *T. gondii* that differ in virulence influence the ability of DCs to cross present parasite derived antigens or act as a source of IL-12. Understanding the mechanics of these events would help in the design of optimal strategies for immune based therapies designed to enhance vaccine-induced responses.

## Materials and Methods

### Mice

DPE-GFP transgenic mice that express GFP on all T cells were originally obtained from Ulrich H. von Andrian (CBR, Harvard, Boston MA) and were crossed to OT1 TCR transgenic mice (The Jackson labs, Bar Harbor, ME) [36],[52]. CD11c^YFP^ transgenic mice were obtained from Michel C Nussenzweig [40]. CD11c-DTR^GFP^ transgenic mice were purchased from the Jackson Laboratory. These transgenic mice were maintained in a specific pathogen-free facility in the Department of Pathobiology at the University of Pennsylvania and the Wistar Institute in conformance with institutional guidelines for animal care. C57BL/6 mice were purchased from the Jackson Laboratory. Mice were used between 6–8 weeks of age and all animal experiments were performed with approval of the Institutional Animal Care and Use Committee (IACUC).

### Parasites

The Prugniaud strain of *Toxoplasma gondii* (Δ HXGPRT) originally obtained from D. Soldtai (Imperial college, London, United Kingdom) [8] was maintained as tachyzoites by serial passage through human foreskin fibroblast cell (HFF) monolayers. Transgenic strains of Prugniaud parasites that were engineered [8] to secrete ovalbumin protein (aa 140–386) into the parasitophorous vacuole (referred to as Pru^OVA^) were maintained similarly on HFF monolayers in the presence of 20 µM chloramphenicol. Pru^OVA^ strains were also engineered to express cytoplasmic dTomato [53] under the control of the alpha tubulin promoter and selected for by phleomycin drug selection. Tachyzoites were purified from the HFF monolayers by filtration through a 3 µm filter (Nucleopore, Clifton, NJ). The parasites were washed, counted and resuspended in PBS for infections.

### Adoptive transfer of OT1 TCR transgenic cells and infections

Lymphocytes were isolated from spleen and peripheral lymph nodes of DPE-GFP OT1 TCR transgenic mice (OT1^GFP^). Single cell suspensions were obtained by mechanical homogenization and RBCs and dead cells were removed by density gradient centrifugation (Lympholyte-M, Cedarlane laboratories Ltd, Hornby, Ontario Canada). T cells were purified using the mouse T cell enrichment columns (R&D systems, Minneapolis, MN). 2×10^6^ purified OT1^GFP^ cells were injected into recipient mice intravenously (retro-orbital injections). 24 hours after transfer of T cells, the mice were infected intraperitoneally with either the parental (Pru) or ovalbumin expressing (Pru^OVA^) prugniaud strains of *T. gondii* at a dose of 10^4^ parasites per mouse.

### Preparation of tissue for live imaging

Mice were sacrificed by CO_2_ asphyxiation and the lymph nodes were removed immediately, with minimal mechanical disruption. They were embedded in 1% agarose (in PBS) in an imaging chamber (Warner Instruments). The embedded lymph node was constantly perfused with warm (37°C) media (RPMI+10% FBS), which was oxygenated (95% O_2_/5%CO_2_). The temperature in the imaging chamber was maintained at 37°C using heating elements and a temperature control probe.

### 2-photon microscopy

Live *ex vivo* imaging was done using a 2-photon microscope system designed by Prairie technology (Ultima) which included a Diode-pumped, wideband mode-locked Ti: Sapphire femtosecond laser (780–980 nm, <140 fs; 90 MHz; Coherent Chameleon), an Olympus BX-51 fixed stage microscope with 20× (NA0.95) or 40× (NA 0.8) water immersion objectives and external non descanned PMT detectors, which consisted of dichroic mirrors (520 nm, 495 nm, and 575 nm) and barrier filters (457–487 nm; 503–537 nm; 525–570 nm and 580–652 nm). In some experiments, a Leica SP5 2-photon microscope equipped with a picosecond laser (Coherent Chameleon; 720 nm–980 nm) and tunable internal detectors that allow simultaneous detection of emissions of different wavelengths and second harmonic signals (SHG) was used. EGFP, YFP and dTomato were excited using laser light of 930 nm. Typically, z stacks of a series of x-y planes at a resolution of 0.49 µm/pixel (40× lens) or 0.98 µm/pixel (20× lens) with a total thickness of 30 µm and step size of 6 µm were captured every 30–45 seconds using the Prairie view acquisition software (Prairie Technologies) or Leica LAS AF software (Leica Microsystems).

### Image analysis

Volocity software (Improvision) was used to convert the three dimensional image stacks into time series. Single cell tracking was done by a combination of manual and automated tracking. For automatic tracking intensity and size filters were used for identifying the cells (exclusion of objects below background intensity levels and size; 50 µm^3^ for T cells and 200 µm^3^ for DCs). The mean migratory velocities, displacement, confinement ratios were calculated using the software. Measurements were typically performed on 31 frames. For measurement of T cell–DC interactions, the duration of contacts of all the T cells observed in a given filed of view were calculated for the entire imaging period (typically lasting 12–15 minutes). Cellular contacts were determined manually as a lack of space between the interacting cells and the data obtained from an average of 6 imaging regions from 3 individual mice per group were used to obtain the frequency of T cells with different durations of contacts with the T cells.

### Parasite DNA estimation by real-time PCR

Parasite DNA levels were measured by real time PCR on DNA isolated from the tissue samples [54]. A 35-fold repetitive *T. gondii B1* gene was amplified by real-time PCR. (Forward primer 5′-TCCCCTCTGCTGGCGAAAAGT-3′; Reverse primer 5′-AGCGTTCGTGGTCAACTATCGATTG-3′). Standard curves for parasite DNA levels were generated using 10-fold serial dilutions of parasite genomic DNA, ranging from 0.1 µg to 0.1 pg, as DNA template. These standard curves were used to measure the total parasite DNA amounts in a given tissue sample.

### RNA isolation and real time PCR for chemokines

RNA was isolated from mesenteric lymph nodes of different immune groups using TRIzol (Invitrogen) and DNase treated total RNA was reverse transcribed using Superscript II (Invitrogen) using standard protocols. Quantitative PCR was performed with customized primer sets for CCL3 and CCL21 (leu) and HPRT (QIAGEN) using Power SYBR green reagents (Applied Biosystems) and an AB7500 fast real time PCR thermal cycler (Applied Biosystems). The values for the CCL3 and CCL21 were normalized to HPRT and displayed as fold induction over naïve controls.

### Isolation of cells from various tissues

Lymph nodes and spleens were isolated from mice at the indicated time points, and single cell suspensions were obtained by mechanical homogenization. The livers were perfused before they were harvested, and lymphocytes were isolated as previously described [55]. For brain mononuclear cells, the mice were perfused with cold PBS, the brain was removed, diced, and passed through an 18-gauge needle and digested with Collagenase/Dispase (25 µg/ml) and DNAse (100 µg/ml) for 45 minutes at 37°C. The cell suspension was then washed and fractionated on a 30%–60% percoll gradient (Pharmacia) for 20 minutes [54]. The cells in the interface consisted of mononuclear cells, which were washed prior to experiments.

### Depletion of dendritic cells

CD11c-DTR^GFP^ transgenic mice were treated with 100 ng per mouse of DT (Diphtheria toxin) IP, to deplete their DCs transiently [6]. Depletion of DCs was confirmed by sampling peripheral blood, 24 hours after DT treatment and staining for DCs by flowcytometry. OT1 T cells were transferred shortly before DT injection and the mice were infected with Pru^OVA^ 24 hours post DT treatment.

### Immuno-histochemistry

Lymph nodes were either directly flash frozen in OCT and or were fixed in 4% formaldehyde/10% sucrose o/n at 4°C (to visualize GFP^+^ cells) prior to freezing. 6 µm sections were immuno-stained as previously described [35] For detection of second harmonic signals, lymph node sections were exposed to polarized laser light (930 nm) on a 2-photon microscope and the signals generated were detected in the 457–487 nm range using barrier filters and non-descanned PMT detectors. All other immuno-histochemical image acquisition and analysis of stained lymph node sections were done using a Nikon fluorescence microscope and the Nikon software (NIS elements).

### Flow cytometric analysis

Freshly isolated cells were stained with the antibodies purchased from eBioscience (San Diego, CA) or BD Biosciences (San Jose, CA) and were run on a FACSCalibur (BD, San Jose, CA). For intracellular staining the cells were stimulated *ex-vivo* for 5 hours in complete media with Brefeldin A, either in the presence or absence of SIINFEKL peptide (1 µg/ml). Following surface staining, the cells were fixed with 4% PFA for 10 minutes at room temperature. Permeablization was done using 0.3% saponin in staining buffer. The cells were run on a FACSCalibur and results were analyzed using FlowJo software (TreeStar Inc., Ashland, OR).

### Statistical analysis

Statistical significance of differences between groups of mice was tested using the student's *t* test or ANOVA/Kruskal-Wallis test for multiple groups. In all the cases, p<0.05 was considered significant.

## Supporting Information

Figure S1 A) Intra-cellular cytokine profile (IFNγ, IL-2, IL-10, TNFα, Granzyme-B, IL-17) of gated CD8^+^ T cells from the lymph nodes of Pru infected mice (day 7 post infection) that were either unstimulated or stimulated with SIINFEKL peptide for 5 hours *ex vivo* in the presence of Brefeldin A. B) Parasite DNA levels (in picograms) in the mesenteric lymph nodes of mice infected with Pru (filled bars) or Pru^OVA^ (open bars) at various time points post infection (36 hours, day 3, day 7 and day 14) is shown. The data are representative of 3 mice per group for each of the time points measured.
(0.28 MB PDF)

Figure S2 A) The co localization of SHG (blue) with ERTR7 (red) on stained 6 µm sections of lymph nodes from day 7 Pru^OVA^ infected mice that had received OT1^GFP^ cells prior to infection. B) Staining for CD11c (red) and ovalbumin (green) on lymph node sections of mice infected with Pru or Pru^OVA^ 36 hours post infection.
(2.77 MB PDF)

Figure S3 A) 3-D rendering of a 30 µm z stack of explanted lymph node from CD11C^YFP^ mice that were infected with Pru^OVA^ dTomato. Arrow points to two adjacent DCs both of which are vacuolated but only one is infected (red parasites).
(0.29 MB PDF)

Video S1 Behavior of OT1GFP cells in Naïve and Pru^OVA^ infected mice. Maximum projection time lapse sequences (30 µm stack) showing the movement of OT1^GFP^ T cells in lymph nodes of uninfected mice (left) and Pru^OVA^ infected mice (day 5 post infection). Time is shown in hh:mm:ss.
(4.68 MB MOV)

Video S2 Changes in morphology of Dendritic cells in response to infection. 3D time-lapse sequences (21 µm stacks) showing the movement of CD11c^YFP^ cells in the lymph nodes of uninfected (left) and Pru^OVA^ infected mice (Day 3 post infection). Note that while the CD11c^YFP^ cells look distinctly different in morphology in the infected animals, no differences in the movement were observed compared to the uninfected animals. Time is shown in hh:mm:ss.
(5.17 MB MOV)

Video S3 Localization of CD11c^YFP^ cells and OT1^GFP^ cells to the sub-capsular region. 3D projections of 150 µm z stacks from uninfected and Pru^OVA^ infected mice showing the localization of CD11c^YFP^ DCs and OT1^GFP^ cells to the sub-capsular region during infection (day 3 post infection). The capsule can be identified based on the second harmonic signals generated and is represented in blue.
(5.71 MB MOV)

Video S4 Vacuolar structures induced within the DCs during infection. 3D projections of 30 µm z stacks from lymph nodes of uninfected (left) and Pru^OVA^ infected (right) mice showing the vacuolated appearance of the DCs induced following infection (day 5 post infection). Also visible is the co-localization of DCs and OT1^GFP^ cells closer to the capsule.
(9.30 MB MOV)

Video S5 Prolonged interactions between DCs and T cells 36 hrs after Pru^OVA^ infection. 3D time lapse sequence of 30 µm stacks showing OT1^GFP^ cells and CD11c^YFP^ DCs making long term contacts in the mesenteric lymph nodes during the first 36 hrs after Pru^OVA^ infection.
(0.78 MB MOV)

Video S6 Short-term interactions between DCs and T cells at day 3 post Pru^OVA^ infection. 3D time lapse sequence of 30 µm stacks showing OT1^GFP^ cells and CD11c^YFP^ DCs making very short or no contacts with one another at day 3 post infection.
(0.92 MB MOV)
