# Supplementary figures and images for: Dynamic Imaging of CD8+ T Cells and Dendritic Cells during Infection with Toxoplasma gondii
Source: PLoS Pathog. 2009 Jul 3;5(7):e1000505. doi: 10.1371/journal.ppat.1000505 (PMC2700268; doi:10.1371/journal.ppat.1000505)

**A**

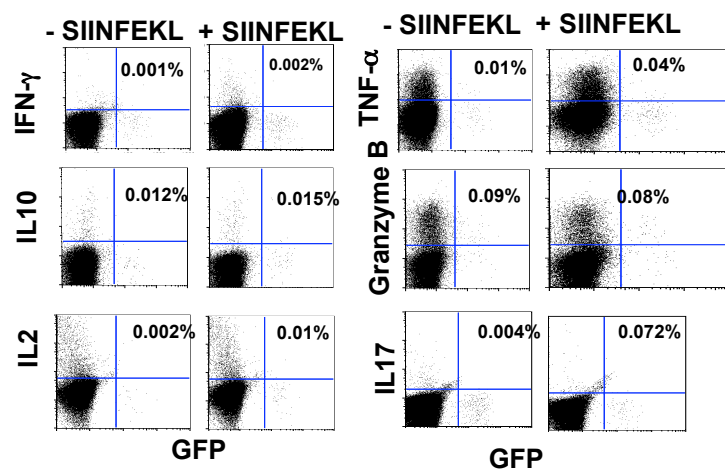

**B**

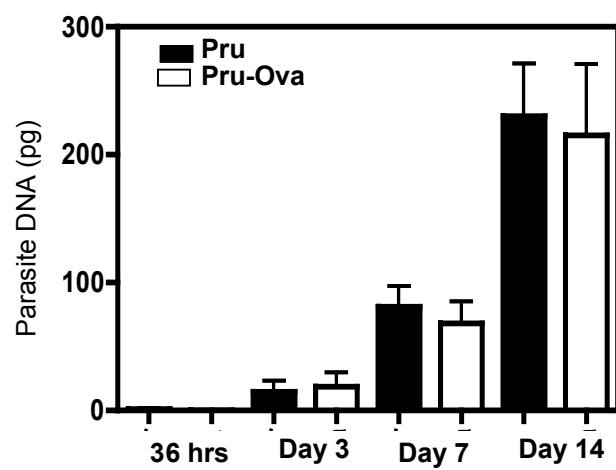

Supplement: Figure S1 — A) Intra-cellular cytokine profile (IFNγ, IL-2, IL-10, TNFα, Granzyme-B, IL-17) of gated CD8+ T cells from the lymph nodes of Pru infected mice (day 7 post infection) that were either unstimulated or stimulated with SIINFEKL peptide for 5 hours ex vivo in the presence of Brefeldin A. B) Parasite DNA levels (in picograms) in the mesenteric lymph nodes of mice infected with Pru (filled bars) or PruOVA (open bars) at various time points post infection (36 hours, day 3, day 7 and day 14) is shown. The data are representative of 3 mice per group for each of the time points measured. (0.28 MB PDF) [file ppat.1000505.s001.pdf]

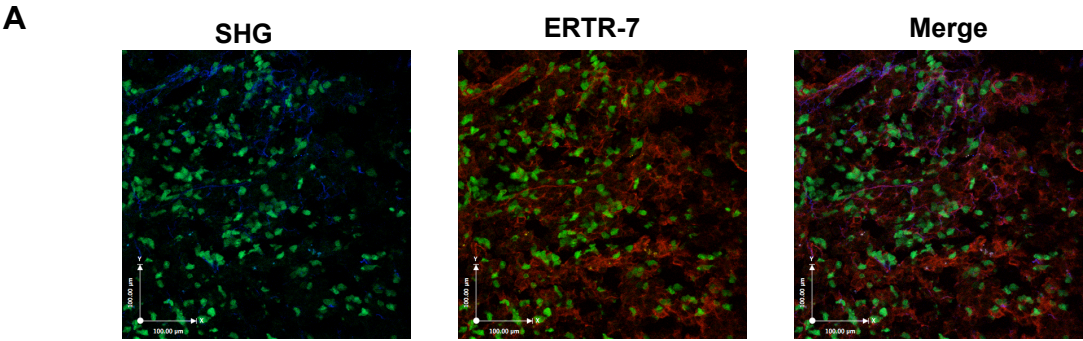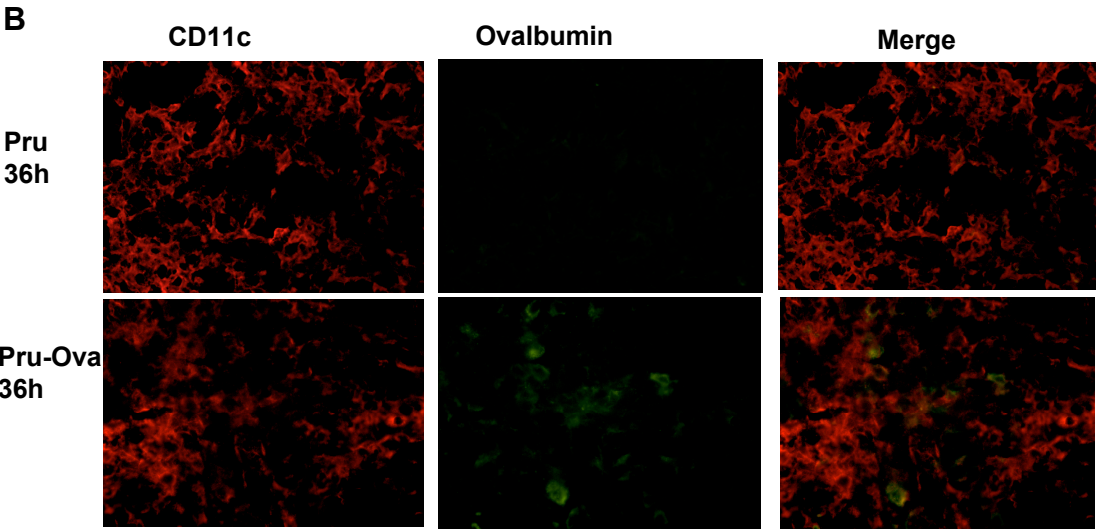

Supplement: Figure S2 — A) The co localization of SHG (blue) with ERTR7 (red) on stained 6 µm sections of lymph nodes from day 7 PruOVA infected mice that had received OT1GFP cells prior to infection. B) Staining for CD11c (red) and ovalbumin (green) on lymph node sections of mice infected with Pru or PruOVA 36 hours post infection. (2.77 MB PDF) [file ppat.1000505.s002.pdf]

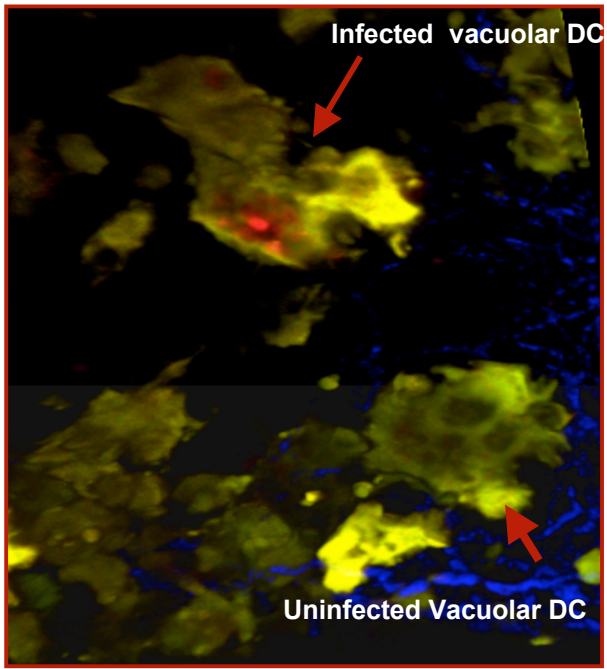

Supplement: Figure S3 — A) 3-D rendering of a 30 µm z stack of explanted lymph node from CD11CYFP mice that were infected with PruOVA dTomato. Arrow points to two adjacent DCs both of which are vacuolated but only one is infected (red parasites). (0.29 MB PDF) [file ppat.1000505.s003.pdf]
